# Making Sense of the 2026 Centers for Medicare and Medicaid Services (CMS) Radiation Oncology Treatment Delivery Codes: Historical Context and Practical Applications for Clinicians

**DOI:** 10.7759/cureus.102412

**Published:** 2026-01-27

**Authors:** Christopher D Jahraus, Dwight E Heron, Tarita O Thomas, Alexander A Harris, Salah Dajani, Teri Bedard, Paul E Wallner

**Affiliations:** 1 Radiation Oncology, American College of Radiation Oncology Advisor to the American Medical Association Relative Value Update Committee, Temple Terrace, USA; 2 Radiation Oncology, Generations Radiotherapy & Oncology PC, Alabaster, USA; 3 Board of Chancellors, American College of Radiation Oncology, Temple Terrace, USA; 4 Oncology, Bon Secours Mercy Health, Youngstown, USA; 5 Radiation Oncology, American College of Radiation Oncology Advisor to the American Medical Association Current Procedural Terminology Committee, Temple Terrace, USA; 6 Radiation Oncology, University of Wisconsin Northern Illinois, Rockford, USA; 7 Radiation Oncology, Radiation Oncology Consultants, Chicago, USA; 8 Radiation Oncology, Essentia Health, Fargo, USA; 9 Coding, Revenue Cycle Coding Strategies, LLC, Austin, USA; 10 Government Relations and Economics Committee, American College of Radiation Oncology, Temple Terrace, USA

**Keywords:** american college of radiation oncology, american medical association, common procedural terminology, general radiation oncology, health policy and economics, medical coding, radiotherapy (rt), specialty society relative value scale update committee

## Abstract

Medical billing and coding remain among the least understood aspects of daily practice for many physicians, including radiation oncologists. Legacy linear accelerator (LINAC)-based radiation treatment delivery codes have remained largely unchanged over the past decade, despite substantial advances in image guidance, intensity-modulated techniques, and motion management. This has drawn the attention of those empowered to insist upon updates to the coding and the amount of time involved in individual therapy administrations. In 2023, the American Medical Association’s (AMA)/Specialty Society Relative Value Scale Update Committee (RUC) Relativity Assessment Workgroup (RAW) mandated re-evaluation of these codes and requested that radiation oncology (RO) specialty societies provide contemporary definitions, times, and resource information for treatment delivery services. The American College of Radiation Oncology (ACRO) and the American Society for Radiation Oncology (ASTRO) jointly developed a new complexity-based code set for megavoltage external beam radiation therapy (EBRT). The RUC subsequently surveyed physicians and recommended relative value units (RVUs). In October 2025, CMS finalized the use of this new code set, effective January 1, 2026. Written by ACRO members involved in the code development and valuation processes, this report places the 2026 code set in historical context and provides practical, physician-focused guidance on applying the new delivery Current Procedural Terminology® (CPT®) codes 77402, 77407, and 77412 (as well as 77387 for professional components of image guidance) in everyday practice. We also summarize the AMA and CMS process, define the complexity framework, and provide concrete clinical examples for appropriate code selection with an emphasis on image guidance, motion management, multiple isocenters, and mixed photon-electron techniques.

## Introduction

A brief history of the current procedural terminology lexicon

As employer-sponsored health insurance expanded rapidly in the post-World War II era, payers lacked standardized definitions for describing medical services, analyzing utilization, and comparing outcomes. This rapid expansion of employer-funded health insurance coverage amplified the problems related to the absence of any standardized medical nomenclature, means of reporting medical record comparisons, statistical evaluation of interventions, and outcomes. The deficiency was especially apparent for surgical services, prompting health insurance providers to approach the American Medical Association (AMA) for a solution. In 1966, in response to this clear need, the AMA published the first edition of Current Procedural Terminology (CPT®) [1].

The first edition was focused on surgical services, with inclusion of some diagnostic and therapeutic procedures, but three subsequent editions published in the 1970s included all of what were then deemed to be standard medical services. The second edition, published in 1970, established the now universally adopted five-digit code format, and in 1983, the Health Care Financing Administration (HCFA), precursor to the Centers for Medicare and Medicaid Services (CMS), adopted CPT as a part of the Healthcare Common Procedure Coding System (HCPCS), the official Federal nomenclature for all medical services and materials [2]. Published annually, the CPT® lexicon now includes more than 11,000 independent descriptors of diagnostic and therapeutic procedures, technologies, and evaluation and management services [3]. Codes are identified in “families” related to specific specialties, but are technically specialty agnostic, so that any Qualified Medical Professional (QMP) who provides the services can appropriately utilize the code(s).

Who owns and maintains the CPT® coding system?

CPT® is a copyrighted, wholly-owned product of the AMA. The AMA Board of Directors (BoD) has entrusted management of the process to the CPT® Editorial Panel (EP), to which it provides staff support. The EP is a 23-member body supported by AMA staff and includes the following: the EP chair and vice-chair, appointed directly by the AMA BoD, 12 members, nominated by medical specialty societies (these societies are selected based on the number of seats in the AMA House of Delegates (HoD)), and one at-large member nominated by other, smaller medical societies. For context, radiation oncology (RO) does not have a seat on the CPT or Specialty Society Relative Value Scale Update Committee (RUC), although the American College of Radiology (ACR) holds a seat representing radiology on both bodies. The Health Care Professionals Advisory Committee (HCPAC) holds two seats, and additional seats are held by nominees of the AHIP (formerly, America's Health Insurance Plans), Blue Cross/Blue Shield Association (BC/BSA), American Hospital Association (AHA), and an umbrella organization of private health insurers. CMS and the US Food and Drug Administration (FDA) hold two seats, both of which give these agencies a voice without voting rights. All EP members, except for those of CMS and the FDA, must be approved by the AMA BoD. The EP is supported in its deliberations by the Specialty Society Advisory Panel ("Advisors"), who represent their individual organizations, provide and comment on applications, and educate their constituents regarding EP decisions.

How are new codes valued?

The RUC was empaneled by the AMA in 1992 to provide input to CMS as it converted from a charge-based payment system to one based on relative values [4]. The committee has evolved to include 32 volunteer members: 22 members are appointed by national medical specialty societies based on their representation in the AMA HoD. The seats are permanent to the societies, with the individuals holding the seats named at the discretion of their respective societies without term limitations; four two-year rotating seats include two from internal medicine-related specialties, one from a primary care specialty, and one from a smaller, otherwise unrepresented society.

The AMA BoD appoints the RUC chair, with the remaining five seats held by a representative of the AMA, the chair of the RUC Practice Expense Subcommittee, the co-chair of the HCPAC, and representatives of the EP and American Osteopathic Association (AOA). Although CMS representatives participate in RUC deliberations, they do not vote on final valuations because all RUC decisions are merely advisory to CMS (and commercial carriers), with all payers able to make independent determinations regarding payment levels. Historically, CMS has accepted greater than 90% of RUC valuation recommendations, though it is under no obligation to do so. Generally speaking, RUC activities, committees, and deliberations are publicly available and are supported by representatives of over 125 medical specialty societies (including American College of Radiation Oncology (ACRO) and American Society for Radiation Oncology (ASTRO) for RO as well as the ACR), who are charged with presenting and defending proposals for their respective specialties and commenting on proposals by others.

Relative value units (RVUs) are based on three main components: physician work (RVU-W), accounting for effort, time, and stress; practice expense (RVU-PE), accounting for staff, equipment, and supplies; and professional liability (RVU-PLI). Within all of medicine, RVU-W averages approximately 51% of the total RVUs, RVU-PE comprises approximately 45%, and RVU-PLI, approximately 4%. For PE-intensive specialties such as RO, the ratio is significantly higher for RVU-PE.

RUC consideration for the valuation of new codes or revision of existing codes is based on surveys from physicians providing the service. For RO, the practice expense is comparatively high given the capital-intensive nature of linear accelerator-based delivery. Methodology for survey development and implementation is strictly controlled by the RUC, including the requirements for the number of valid surveys: if a service is provided < 100,000 times/year, a minimum of 30 valid surveys is required. The RUC considers all codes on a five-year rolling basis, and new or revised codes referred to it from the CPT® EP, but will also review codes targeted for review by its own Relativity Assessment Workgroup (RAW), CMS, or other appropriate entities. Consequently, the RUC relies on these tightly controlled physician surveys to quantify the time and intensity of specific services. CMS has historically adopted the majority of RUC recommendations but may independently adjust valuations, especially practice expense, to maintain budget neutrality and consistency with the Hospital Outpatient Prospective Payment System (HOPPS) [5].

In 2023, the RAW determined that linear accelerator (LINAC)-based radiotherapy delivery codes were potentially over-valued and out of sync with contemporary practice and referred them back to the CPT® EP for redefinition. It was for this reason, and concerns regarding misuse of superficial radiation therapy (SRT) codes, that RO societies collaborated to redefine those codes.

Did organizations representing RO request new codes?

Particularly pertinent to the 2026 code changes, the EP may consider proposals for new codes, changes in existing codes, where interventions have changed over time, or where the existing definitions seem to inadequately define the service as currently provided. Although applications for code consideration in the form of a Correct Coding Application (CCA) may come from any source, such as direct referral, at a historical minimum, support of a medical specialty society is an effective requirement. Following completion of a code definition or revision, the EP will refer the new code(s) to the RUC for valuation. At its discretion, the RUC may refer a code back to the EP if it determines that valuation is impossible or inappropriate based on a specific definition or interpretation of that definition. CCA proposals from specialty societies and/or vendors always have economic implications, including projections on frequency of use, but EP deliberations are payment agnostic.

In a “revenue-neutral” milieu, where gains in one code or code set have negative implications for all other codes, RUC deliberations have no similar preclusion. Although the EP publishes its final code definitions, full CCAs, and deliberations related to the applications remain confidential to EP members, panel advisors, and staff, in this, the processes are balanced to maintain transparency while allowing for candid discussion amongst the panel members. In the case of the 2026 treatment delivery codes, both the ACRO and ASTRO requested that no changes be made to the code set in 2023, but the RUC determined that a new code set must be developed, and the process of developing the codes discussed here was started.

## Technical report

From legacy G-codes to a complexity-based CPT structure

Since 2015, LINAC-based megavoltage treatment delivery in the Medicare Physician Fee Schedule (MPFS) has been reported primarily with a series of G-codes (G6003 to G6016), which were listed by CMS in response to congressional action just before the time of their inclusion [6]. Separately, image-guidance services were reported with 77014 (most typically cone-beam CT) or other imaging codes (G6001, G6002, and G6017). It is important to note that the G-codes were established as a workaround implemented in 2015, not as standard CPT® descriptors. They could not be handled through the usual CPT® EP process without deliberate transition. 

In September 2023, the RAW concluded that the G-codes required re-evaluation, but because they were not CPT® codes, it referred the matter to the CPT® EP for full revision and consolidation into the CPT® ecosystem. The use of these non-standard CMS-issued codes was the result of legislative action that prevented the use of the proposed AMA-devised codes for radiotherapy delivery.

Development of the new code set

Over the following year, representatives of ACRO and ASTRO collaborated to devise a new set of codes, reflecting the definition of radiotherapy delivery (including image guidance when used) according to complexity. In 2024, the CPT® accepted the new code definitions proposed by ACRO and ASTRO, and the societies collaborated again to survey ROs regarding the time involved in the delivery of each of the services described.

At the January 2025 meeting of the RUC, values were proposed based on data from practices responding to the survey, and the number of RVUs per service was thereafter recommended to CMS. CMS issued its proposed rule in July 2025 and chose to disregard aspects of the AMA valuation, instead basing the PE components of treatment delivery on factors common to the HOPPS expense determinations. The societies recommended modifications to some aspects of code valuation, and in October 2025, CMS issued the MPFS final rule implementing the new code definitions as proposed, with values and RVU components determined by CMS. The codes, their descriptions, and associated payment approximations are detailed herein. 

In the Discussion section, we offer practical guidance regarding examples of what we believe constitutes appropriate use of each code. None of our statements should be construed as "rule-making" as the authors neither have nor claim such authority. We offer our interpretation as some of the original writers involved in the process. We stress that these coding changes only impact radiotherapy treatment delivery codes, including image guidance, but not treatment planning, management, and other associated radiotherapy service codes. We also note that superficial radiotherapy, the vast majority of which is delivered by dermatologists, now has entirely separate code definitions, and these codes are beyond the scope of this manuscript.

Services represented by the 17 different G-codes and by code 77014 (for cone-beam CT-based image guidance) have been removed from the coding lexicon and replaced with four newly revised CPT® codes: 77387, 77402, 77407, and 77412 [7]. The result is a streamlined four-code structure for megavoltage external beam radiation therapy (EBRT):

77387

This is the professional component of all forms of image guidance, which does not include motion management. The technical components of image guidance and, when appropriate, active motion management, are included in the three remaining treatment delivery codes [7].

77402 (Level 1)

Simple 2D or electron beam delivery. This generally involves simple setups, including simple *en face* electron fields. Although image guidance would be included in this delivery code, plans actually requiring image guidance would not typically be coded with 77402 [3].

77407 (Level 2)

This concerns three-dimensional conformal radiation therapy (3D-CRT) or intensity-modulated radiation therapy (IMRT)/volumetric modulated arc therapy (VMAT), single isocenter, no motion management [7]. 

77412 (Level 3)

This concerns treatment requiring motion management and/or multiple isocenters and/or mixed photon-electron or total skin electron therapy. It is critical to note that application of code 77412 does not require the use of any particular system for active motion management, is not vendor-specific, and each of the four criteria described is sufficient, each on its own, for delivery of therapy under this code. For clarity, multiple criteria are not required to constitute treatment with the code [7].

Stereotactic radiosurgery (SRS) and stereotactic body radiation therapy (SBRT), treatment planning and management codes were not substantially altered for 2026 and are therefore also not the focus of this report.

## Discussion

Each of this report’s authors was part of the ACRO team involved in developing and establishing values for the new codes. As such, we hope to offer insights into the thought processes behind each code, as that relates to the proper use of the new code set. We are quick to emphasize that the new codes, which we discuss, only apply to megavoltage photon and electron radiotherapy delivery (including image guidance) and are separate from the newly devised superficial radiotherapy coding, used principally by dermatology practices delivering radiotherapy. 

Radiotherapy treatment planning and management, as well as stereotactic radiotherapy coding has not been substantially changed for 2026. Additionally, both ACRO and its colleague organizations advocate for the sustainable reimbursement of radiotherapy via whatever codes are developed. It is ultimately CMS that determines what will be paid for under the Medicare fee schedules. We are pleased to note that CMS has seen the merit of aligning billing rules and cost bases between the HOPPS and MPFS, and we hope to see even greater site-neutrality in the future [8]. We discuss each code’s implications in numerical order.

77387 image guidance

CPT® code 77387 is used to report the acquisition, fusion, review, and interpretation of image guidance for the localization of the target volume for the delivery of radiation treatment. Historically, CPT® code 77387 represented both the technical and professional aspects of image guidance, or it only represented the professional component with the addition of modifier 26. For 2026, CMS labelled the technical component of CPT® 77387 as a “B” status code, meaning it will be bundled and no longer billed and paid separately. This keeps it consistent with the HOPPS rule, where the technical part of 77387 is included in the payment for other treatment delivery codes (77402, 77407, and 77412, to be discussed below).

While the technical component of this code will be bundled, the professional part will still be billed separately. Therefore, effective January 1, 2026, 77387 will represent the professional component of treatment localization utilizing image guidance, previously represented by 77387 with modifier 26 under HOPPS or using 77014 (and certain image guidance other codes) under the MPFS. 

In parallel with the definitional revision, 77387 required a new RUC survey, leading to a reassessment of its physician work value. The code received a total of 102 survey responses with a new RVU of 0.70, representing a reduction from the 0.85 RVU previously assigned. An efficiency adjustment was applied during the valuation process, resulting in a finalized RVU of 0.68 in the MPFS final rule.

77402 Level 1 radiation treatment delivery

CPT® code 77402 is used to report the simplest form of radiation treatment delivery. This includes delivery with a single electron field, multiple non-abutting electron fields, or two-dimensional (2D) photon treatment. It should be noted that utilization for 77402 is projected to be generally less than 5% of all radiation therapy treatment delivered. 

Cases appropriate for 77402 would include simple skin cancer treatments with an *en face* electron beam. For example, a patient might come in with either a single or multiple skin cancers on different anatomical locations that could be treated as a single treatment administration, coding with 77402. 

An example scenario for a simple 2D plan might involve the use of hand calculations for an emergency (“sim and treat”) spine treatment using a single PA field; however, we would not expect typical spine treatments to fall under this category. In the example of an emergency situation, one might subsequently create a more refined plan one or two treatments into the process, when greater resources are available. Such refined plans would not fall under 77402, as they would typically involve traditional isocentric treatment with planning appropriate to minimize dose to normal tissues (e.g., the esophagus in thoracic spine treatment or bowel in lumbar spine treatment). For clarity, these situations, which typically necessitate wedges, field segmentation, and other characteristics of 3D treatment planning, would rise to the level of 77407. 

We emphasize what is not considered Level 1 therapy (i.e., that which rises to a code higher than 77402). With that in mind, IMRT is not included as a treatment modality represented by 77402, just as a 3D planned treatment utilizing multiple fields, with blocks or segments is not included either. Another example of treatment that should not be mistaken for Level 1 would be whole brain radiation treatment with eye blocks and neck blocks. This, too, would be considered 3D planning. Although image guidance can be utilized for this type of low complexity radiation therapy based on the need for accurate treatment delivery, we struggle to envision common scenarios in which a simple treatment would necessitate image guidance. 

For calendar year 2026, under the HOPPS fee schedule, CPT® code 77402 will be assigned APC level 5621 with a payment rate of approximately $108 and status indicator S (procedure or service not subject to multiple procedure discounting).

77407 Level 2 radiation treatment delivery

By most estimations, CPT® code 77407 will be the “workhorse” of radiotherapy delivery codes. CMS estimates that greater than 50% of radiotherapy delivery will fall into this category. As such, it includes all photon radiotherapy delivered to a single isocenter using either 3D conformal radiotherapy planning techniques or IMRT techniques, but not requiring any form of active motion management. Within the scope of 3D conformal treatment planning, we include segmented fields used to minimize dose heterogeneity (sometimes called “field-in-field” techniques), wedges to improve dose heterogeneity, and the use of complex blocking to avoid normal tissues. If even a single normal tissue dose constraint must be met, then the treatment is by definition one that necessitates 3D planning and assessment of a dose volume histogram (DVH). We offer numerous examples, ranging from relatively simpler 3D plans to those that are quite complex, but do not necessitate the use of active motion management, nor multiple isocenters, nor mixed electron-photon techniques (as these exceptions would all qualify as Level 3 therapy using code 77412). 

A simple example of a case appropriate for the use of 77407 would be an isolated metastasis to the iliac wing. Given the proximity of the iliac wing to normal bowel, 3D planning to assess bowel dose is appropriate. Beyond that, the shape of the anatomical structure around the bone (e.g., the buttocks) may necessitate the use of wedges to achieve optimal dose homogeneity. Posteriorly located structures such as this generally do not move with respiration, and therefore active motion management would not typically be required; hence, this is a simple, but appropriate case for 77407 (Level 2). A similar situation would apply to low thoracic and upper lumbar spinal bone metastases. In this case, one might seek to minimize cardiac and/or esophageal dose using an array of posterior beams, rather than a simple anteroposterior (AP)/posteroanterior (PA) set-up. While such treatment certainly rises to the level of 3D planning, it would not likely require active motion management and is therefore appropriate for billing with 77407. Of note, we believe a majority of other sites within the thorax and abdomen (ribs and soft tissue sites) move with respiration, and as such, 77412 treatment, including active motion management, is appropriate.

Comparatively more complex scenarios in which 77407 is appropriate would include treatment of prostate cancer, and indeed, this is one of the clinical vignettes that was used to illustrate the coding structure. Particularly when it involves elective treatment of lymph nodes, this requires complex IMRT planning; however, the prostate and nodes undergo little intra-fraction motion, and to our knowledge, there are not commonly used systems that would track such motion during administration of a single treatment fraction. Similarly, radiotherapy for head and neck cancer is among the most complex of treatment designs, and yet its lack of need for active motion management means that 77407 will typically be the most appropriate code. The exception to this, in the case of cancers of the head and neck, might be those in which electron and photon radiotherapy are combined in each individual treatment delivery, as mixed electron-photon techniques are considered Level 3 and would be coded with 77412.

The most common reason for which a treatment would go beyond 77407 into the 77412 level of complexity will be the need for motion management. This is discussed further below.

77412 Level 3 radiation treatment delivery

CPT® code 77412 was revised for 2026 to account for the changes in complexity and practice patterns for EBRT. These changes account for the greater treatment time utilized with complex treatments. The utilization expectations for code 77412 are moderate to high, estimated by CMS to be roughly 35% of EBRT treatments [8]. Level 3 EBRT is not intended to be the most utilized level of treatment delivery; therefore, understanding when it is applicable is key to ensuring proper coding. 

The following are the key differences that separate Level 3 from Levels 1 and 2 for EBRT. Note that any one of the following four criteria is adequate to meet coding guidelines for 77412: (i) Use of active motion management, which includes surface guidance, active breathing control, deep inspiratory breath hold, four-dimensional (4D) cone-beam CT (CBCT, done immediately before treatment), and potentially other means; (ii) Multiple isocenters planned with 3D or IMRT; (iii) Mixed modality of photon and electron fields; (v) Total skin electron therapy

Understanding the proper use of code 77412 necessitates an understanding of certain definitions.

Level 3 criterion: active motion management

Active motion management involves intra-fraction localization and/or tracking of the target, organ-at-risk (OAR), or patient motion during beam delivery, and is specific to the time the beam is on, and/or daily analysis of or limiting patient motion, and delivering radiation to the patient. This may involve the use of implanted fiducial markers and imaging to monitor beam delivery, but does not require fiducials specifically. It goes beyond simple setup verification or a tattoo-less position. Typical tools include: (i) Respiratory-gated delivery with external surrogates or internal markers; (ii) Deep inspiration breath-hold (DIBH) systems for breast, thoracic, and upper abdominal treatments; (iii) Surface-guided radiotherapy systems providing real-time monitoring, when surface motion correlates with tumor motion; (iv) 4D CBCT or similar systems used to adjust for intra-fraction motion.

Multiple studies have quantified respiratory motion amplitudes on the order of 5-20 mm for lung and liver tumors with potential for substantial target displacement and normal tissue irradiation if unmanaged. Motion-guided techniques reduce margins, improve target coverage, and can lower the doses to nearby OARs.

Our team realizes that an oft-cited reason for third-party payers denying authorization for advanced techniques like this is a lack of prospective data, which proves such interventions improve the outcome of patients compared to treatment without such methods. It is important to note that numerous studies have described advantages to the use of motion management in a variety of treatment scenarios [9-12]; however, we believe that it could be potentially unethical to perform a randomized controlled trial of motion management vs. no motion management, when treatment volumes can clearly be reduced with its use based on dosimetric data alone. Therefore, it seems highly unlikely there will ever be a prospective randomized controlled trial of motion-managed treatment of any particular site vs. non-motion-managed treatment. Logic and common sense must prevail in allowing motion management for any target that moves with respiratory excursion. To that end, our team believes that a majority of treatments in the chest and abdomen warrant consideration of active motion management, with the potential exception of immobile posterior treatment sites such as the spine. It is possible that in the future, motion-managed approaches for other sites will become standard as well, though at this time, management of respiratory excursion is the most widely used approach. We offer examples of appropriate use cases for 77412 below.

Left or right-sided breast cancer radiotherapy is an obvious example. Consider a patient whose therapy was devised with a single isocenter dosimetry treatment plan for the treatment of the whole breast and supraclavicular nodes. During treatment delivery, the patient will be treated with the use of active motion management to monitor the target and organs at risk (or facilitating their immobilization) during the treatment delivery. Although there is a single isocenter for this course, the use of daily active motion management to monitor motion during the breathing cycle supports code 77412. It is critical to note that active motion management does not consist of any particular manufacturer’s system or technology. In general, we would include any of the following to be active motion management, and indeed, each of these was mentioned in the code development process: deep inspiratory breath hold (using a commercially available system), respiratory gating, surface guided radiotherapy, and 4D cone-beam CT, as well as potentially others. 

Level 3 criterion: multiple isocenters

An isocenter is that point around which an isocentric treatment accelerator’s components rotate. It is specified in the dosimetry treatment plan as a reference point for the treatment machine coordinate system. Multiple isocenters are required when treating geographically separate targets that cannot be encompassed safely or practically under a single isocenter. Examples include: (i) Simultaneous treatment of humerus and femur metastases; (ii) Concurrent treatment of brain and lung metastasis in the same session; (iii) Extended-length treatments such as craniospinal irradiation, and fields of a length that exceeds the physical limits of a single field, as may be the case in treatment of a femur.

Although each individual field arrangement may be relatively straightforward, the additional setup, positioning, and imaging time for each isocenter may justify a Level 3 selection. Thus, when a patient is treated to more than one distinct treatment volume (e.g., lung and brain), 77412 treatment delivery is supported. Of note, changing isocenters (as one might do between the initial and boost phases of an overall plan) does not constitute multiple isocenters, in that only one isocenter is used per treatment day.

Level 3 criteria: mixed photon-electron treatment and total skin electron therapy (TSET)

Mixed modality treatment involving both photon and electron fields for the same volume inherently introduces additional complexity because they require: (i) Separate isocentric (photon) and visual or surface-based (electron) setup; (ii) Distinct depth-dose characteristics that must be carefully coordinated to cover superficial and deep components of a target volume. This additional time and complexity warrants use of 77412 treatment delivery.

One can conceive of numerous scenarios in which a neglected tumor, such as a breast cancer, spans a broad range from erosion of the skin surface to a depth of 5 cm or more. For such situations, the reliable application of tissue equivalent bolus material may be impractical, due in part to the irregularities of the skin surface eroded by the neoplasm. In such situations, a 6 MV photon beam deposits only about 50% of the maximum depth dose (dMax) at the skin surface, and about 85% of the dMax at a depth of 5 mm in tissue. Clearly, there is an advantage to the application of electrons, as in this case, using a 15 MeV electron beam delivers just over 90% of the dMax dose to the surface; however, this falls to just under 80% at 5 cm depth in tissue. This is an ideal case for mixed photon-electron treatment. Here too, the treatment plan requires fundamentally two setups, one isocentric for the photon component of treatment and one visual for the electron component. This photon-electron combination treatment qualifies as a Level 3 treatment, and 77412 is appropriately used.

Total skin electron therapy is among the most laborious of all treatment deliveries, often involving multiple patient positions, extended quality assurance efforts, and was recognized by the code development team, constituting 77412 treatment delivery

High-yield facts

Appropriate use of the 2026 treatment delivery codes includes the following: (i) If treatment requires volumetric planning & normal tissue constraints, it constitutes at least Level 2 (77407). If it additionally requires active motion management, multiple isocenters, or mixed modality, it rises to Level 3 (77412). (ii) There is a distinction between IGRT and active motion management. For active motion management (criteria for 77412), continuous or gated control of intra-fraction motion during beam delivery is required. Conversely, IGRT (77387) is image-based localization and verification before or around the time of treatment. The exception to this would be 4D CBCT, in which the motion is analyzed daily and compared to the motion assessed at simulation. (iii) A conventional CBCT taken before treatment and reviewed by the physician justifies 77387 (professional component) and, depending on the plan complexity, 77407 or 77412. IGRT alone does not justify the use of 77412. (iv) Documentation is essential and should clearly support the use of the selected treatment delivery codes.

**Figure 1:**
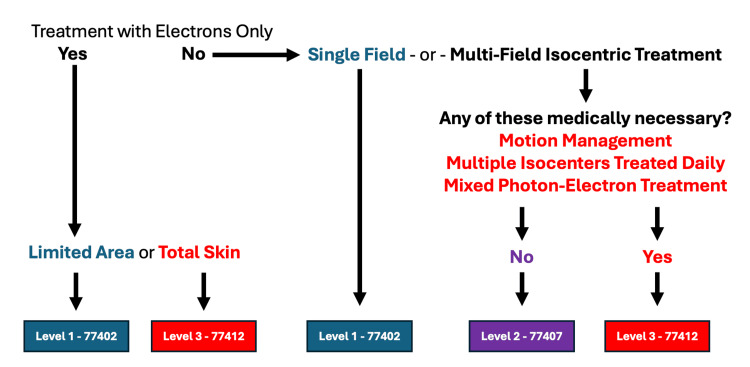
2026 Megavoltage Radiotherapy Delivery Coding Image Credit: Christopher D. Jahraus The American Medical Association (AMA) holds the copyright to the Current Procedural Terminology (CPT®) codes.

A decision tree to assist in optimal code determination is shown in Figure 1. It is to be noted that there may be exceptions to this in individual circumstances.

## Conclusions

Herein, we have presented a detailed summary of changes to radiotherapy treatment delivery codes for 2026. Again, we highlight that the coding structure for megavoltage radiotherapy planning, management, and associated services has remained largely unchanged. While it is impossible for any single report to specify the optimal use of the 2026 radiotherapy treatment delivery code set in every situation, we nonetheless empaneled a group of experts involved in writing the code descriptions and defining the value of each. We offered specific examples of appropriate use of each code and provided objective guidance for proper treatment delivery coding.

Ultimately, each physician must consider the unique scenarios they encounter and determine appropriate coding by the most objective means possible. As technology continues to evolve, particularly as it relates to adaptive radiotherapy, MRI-guided workflows, and artificial intelligence-driven motion management, collaborations between specialty societies, the AMA, CMS, and other stakeholders will be crucial to ensure that coding and reimbursement remain aligned with contemporary clinical practice.
